# Supplementary material for: Cost-effectiveness of point-of-care C-Reactive Protein test compared to current clinical practice as an intervention to improve antibiotic prescription in malaria-negative patients in Afghanistan
Source: PLoS One. 2021 Nov 8;16(11):e0258299. doi: 10.1371/journal.pone.0258299 (PMC8575266; doi:10.1371/journal.pone.0258299)
Supplement: S2 Appendix — (DOCX) [file pone.0258299.s002.docx]

# S2 Appendix: model parameters and sources

| **Parameter** | **Parameter Description** | **Population (patients)** | | | | | |
| --- | --- | --- | --- | --- | --- | --- | --- |
| Pop | Population | 4391 | | | | | |
| **Cost Parameter** | **Parameter Description** | **Mean**  **($)*** | **SD**  **($)** | **95% CI** | **Distribution (α, β) ^+^** | **Source** | **Comment** |
| cTEST | Cost of the POCCRP test kit | 1.00 | 0.20 | 0.61 - 1.39 | Gamma (25, 0.04) | (26) | Conservative estimate used to allow for added costs for import tariffs, shipment, and other peripheral expenses (26). |
| cTRAIN | Training to administer and interpret test | 0.03 | 0.01 | 0.02 - 0.04 | Gamma (25, 0.001) | (40) | Assumption cost of training for a POCCRP test would be the same as malaria RDT. |
| cOPD | Outpatient services delivery cost | 0.28 | 0.06 | 0.25 - 0.31 | Gamma (376.83, 0.001) | (40) | Average taken across the 22 healthcare facilities in Afghanistan. |
| cDISP | Overhead cost of running the pharmacy/dispensary at the healthcare facilities | 1.33 | 0.10 | 1.29 - 1.38 | Gamma (2.31, 0.58) | (40) | Average taken across the 22 healthcare facilities in Afghanistan. |
| cOPEapp | Patient out of pocket expenditure when patient receives appropriate treatment. (Includes cost of transport, drugs, consultation fees, laboratory fees, or special food) | 3.82 | 12.32 | 2.95 - 4.65 | Gamma (51.64, 33.61) | (40) | “Household costs were captured in a sample of 676 suspected malaria patients (508 in moderate transmission region; 168 in low transmission region) for a 28-day period starting from the day a patient first sought care at one of study health centre.” (40)  “All interviews inquired if the fever illness had resulted in days where the patient and the main caregiver were unable to perform their normal activities and how much time had been spent travelling to and waiting at health providers. This opportunity cost of time lost was valued at GDP per capita per day in 2009” (40).  Average cost used between the two regions for six household cost inputs; weighting applied based on the sample proportion between regions.  Costs could not be disaggregated in the primary data between malaria-positive and malaria-negative patients. |
| cPLTapp | Opportunity cost of patient’s time when patient receives appropriate treatment | 3.20 | 4.47 | 2.89 – 4.74 | Gamma (0.51, 0.07) | (40) |  |
| cCLTapp | Opportunity cost of carer’s time when patient receives appropriate treatment | 1.44 | 2.71 | 1.24 – 1.65 | Gamma (151.73, 0.009) | (40) |  |
| cOPEnot | Patient out of pocket expenditure when patient receives inappropriate treatment. (Inclusions same as cOPEapp) | 5.61 | 17.16 | 4.32 - 6.91 | Gamma (14.77, 0.38) | (40) |  |
| cPLTnot | Opportunity cost of patient’s time when patient receives inappropriate treatment | 4.15 | 4.90 | 3.78 - 4.52 | Gamma (99.01, 0.04) | (40) |  |
| cCLTnot | Opportunity cost of carer’s time when patient receives inappropriate treatment | 1.85 | 2.42 | 1.67 – 2.03 | Gamma (80.67, 0.02) | (40) |  |
| cANTI | Cost per full course of antibiotics in Afghanistan | 0.12 | 0.02 | 0.11 – 0.12 | Gamma (25, 0.005) | (40) | Full course defined as 10 standard units. |
| cAMR | Cost of AMR to society per full course of antibiotics | 4.39 | 0.88 | 2.67 – 6.11 | Gamma (25, 0.18) | (41) | In the source study for this parameter “the economic cost of AMR is narrowly defined as the incremental cost of treating patients with resistant infections as compared with sensitive ones, and the indirect productivity losses due to excess mortality attributable to resistant infections”. The study considers cost of AMR in 5 pathogens.  Estimate for a broad-spectrum penicillin (BSP), assumed to drive resistance in 5 pathogens in Thailand. BSP chosen as most likely to be used as first line of antibiotic treatment in Afghanistan. Full course defined as 10 standard units. |
| **Probability Parameter** | **Parameter Description** | **Probability*** | **95% CI** | | **Distribution (α, β) ^+^** | **Source** | **Comment** |
| pBac | Prevalence of bacterial infection among malaria-negative patients | 0.10 | 0.09 – 0.10 | | Beta  (15, 142) | (13) | Aetiology of undifferentiated fever is acknowledged as an area that requires further research (12,15,42). There is a limited evidence base for prevalence of bacterial infection in non-malaria febrile patients in various countries (14,36–39). Data from Afghanistan was provided by researchers of an unpublished study (13). |
| pDIAGanti | Probability of being prescribed antibiotic under current clinical practice | 0.56 | 0.56 – 0. 56 | | Beta  (2479, 1912) | (30) | Calculated as proportion of patients from the population under clinical practice who were prescribed an antibiotic. The model assumes that given the difficulties in differentiating a bacterial infection from others causes (43), a healthcare worker would diagnose the same proportion of patients with an antibiotic when they have a bacterial infection and when there is another cause. |
| pSENSpoccrp | Sensitivity of POCCRP test for detection of CRP concentration at 10 mg/L | 0.95 | 0.87 - 0.97 | | Beta  (86, 5) | (21) | Probability that the POCCRP will provide positive result if there is a CRP concentration of 10 mg/L or more. |
| pSPECpoccrp | Specificity of POCCRP test for non-detection of CRP concentration of less than 10 mg/L | 0.98 | 0.92 - 1.00 | | Beta  (75, 2) | (21) | Probability that the POCCRP will provide negative result if there is a CRP concentration of less than 10 mg/L. |
| pBACcrp>10 | Probability of bacterial infection patient having CRP concentration of 10 mg/L | 0.95 | 0.92 - 0.97 | | Beta  (404, 21) | (20) | Probability that a bacterial infection patient will have a CRP concentration of 10 mg/L or more. |
| pOTHcrp<10 | Probability of non-bacterial infection patient having CRP concentration of less than 10 mg/L | 0.49 | 0.46 - 0.53 | | Beta (464, 483) | (20) | Probability that a non-bacterial infection will have a CRP concentration of less than 10 mg/L. |

* Value used for the deterministic model

**^+^** Distribution for PSA

**Note for S2 Appendix:**

Alpha (α) and Beta (β) values for Gamma distributions calculated as below (where $\bar{x}$ denotes mean and σ_x̅_ denotes standard error):

${\alpha=\left( \frac{\bar{x}}{\sigma_{\bar{x}}} \right)}^{2}$

$\beta=\frac{{\sigma_{\bar{x}}}^{2}}{\bar{x}}$

Alpha (α) and Beta (β) values for Beta distributions are assigned based on counts of events of interest as a proportion of total sample. Standard error (σ_x̅_) is calculated as below:

$\sigma_{\bar{x}}=\sqrt{\frac{\alpha\beta}{({\alpha+\beta)}^{2}+(\alpha+\beta+1)}}$
