# Supplementary figures and images for: Cost-effectiveness of point-of-care C-Reactive Protein test compared to current clinical practice as an intervention to improve antibiotic prescription in malaria-negative patients in Afghanistan
Source: PLoS One. 2021 Nov 8;16(11):e0258299. doi: 10.1371/journal.pone.0258299 (PMC8575266; doi:10.1371/journal.pone.0258299)

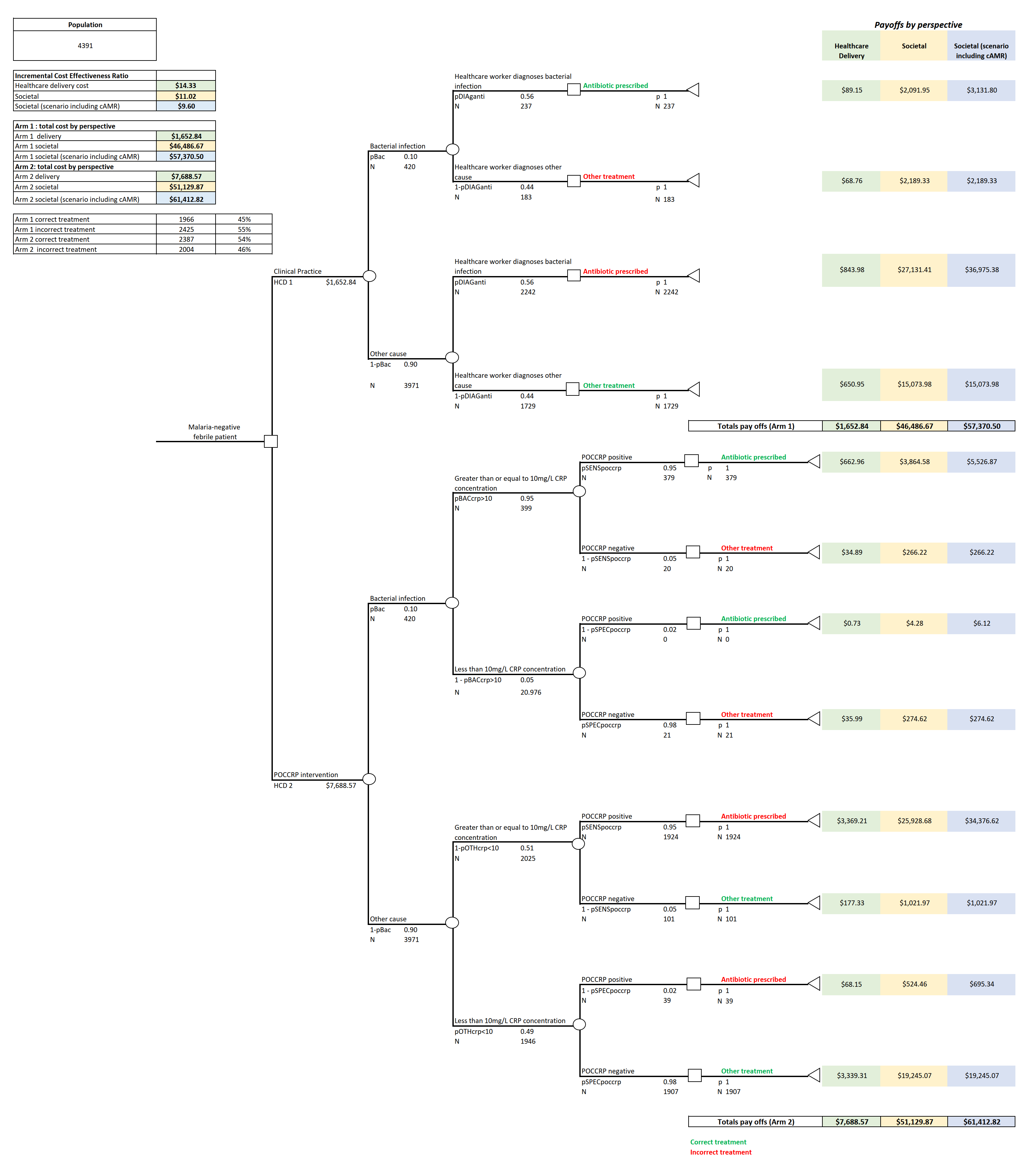

Supplement: S3 Appendix — (TIFF) [file pone.0258299.s003.tiff]
