# Supplementary material for: Cost-effectiveness of point-of-care C-Reactive Protein test compared to current clinical practice as an intervention to improve antibiotic prescription in malaria-negative patients in Afghanistan
Source: PLoS One. 2021 Nov 8;16(11):e0258299. doi: 10.1371/journal.pone.0258299 (PMC8575266; doi:10.1371/journal.pone.0258299)
Supplement: Attachment — Submitted filename: Response to Reviewers.docx [file pone.0258299.s004.docx]

Simon Dickinson

24 Borough Road

Altrincham

WA15 9RA

14 September 2021

PLOS ONE, 1265 Battery street, Floor 2,

San Francisco, CA94111, USA

**Subject: Response to comments on journal article: “Cost-effectiveness of point-of-care C-Reactive Protein test compared to current clinical practice as an intervention to improve antibiotic prescription in malaria-negative patients in Afghanistan”.**

Dear Editor,

Thank you for sharing your comments, as well as those of the three reviewers. This letter describes the changes we have made to our manuscript in response to the comments provided by the reviewers, the academic editor and more general points with regard to format, funding statement and competing interest statement. We thank the reviewers for their comments, which have undoubtedly improved the quality of the manuscript. In line with the guidance provided, this letter is also accompanied by:

- a marked-up copy of the manuscript that highlights changes made to the original version, labelled 'Revised Manuscript with Track Changes'.
- an unmarked version of your revised paper without tracked changes, labelled 'Manuscript'.
- a completed CHEERS checklist (as recommended by Academic Editor).
- a revised version of Fig 7 (as recommended by Academic Editor).

## Response to PLOS ONE general points (not related to article content)

| **Comment** | **Response** |
| --- | --- |
| 1. Please ensure that your manuscript meets PLOS ONE's style requirements, including those for file naming. The PLOS ONE style templates can be found at  <https://journals.plos.org/plosone/s/file?id=wjVg/PLOSOne_formatting_sample_main_body.pdf> and  https://journals.plos.org/plosone/s/file?id=ba62/PLOSOne_formatting_sample_title_authors_affiliations.pdf | We have updated the manuscript based on the style guide provided. |
| 2. Thank you for stating the following in the Financial Disclosure section:  "The authors received no specific funding for this article. The primary data collection (2009 - 2012) for the malaria RDT IRT (30) and CEA (41) in Afghanistan was funded by the Bill and Melinda Gates Foundation through a grant to the London School of Hygiene and Tropical Medicine. Data on aetiological agents of disease (14) was collected with funding from the UK Defence Science and Technology Laboratory."  We note that one or more of the authors are employed by a commercial company: "Mott MacDonald Ltd;"   a) Please provide an amended Funding Statement declaring this commercial affiliation, as well as a statement regarding the Role of Funders in your study. If the funding organization did not play a role in the study design, data collection and analysis, decision to publish, or preparation of the manuscript and only provided financial support in the form of authors' salaries and/or research materials, please review your statements relating to the author contributions, and ensure you have specifically and accurately indicated the role(s) that these authors had in your study. You can update author roles in the Author Contributions section of the online submission form.  Please also include the following statement within your amended Funding Statement.  “The funder provided support in the form of salaries for authors [insert relevant initials], but did not have any additional role in the study design, data collection and analysis, decision to publish, or preparation of the manuscript. The specific roles of these authors are articulated in the ‘author contributions’ section.”  If your commercial affiliation did play a role in your study, please state and explain this role within your updated Funding Statement.   b) Please also provide an updated Competing Interests Statement declaring this commercial affiliation along with any other relevant declarations relating to employment, consultancy, patents, products in development, or marketed products, etc.  Within your Competing Interests Statement, please confirm that this commercial affiliation does not alter your adherence to all PLOS ONE policies on sharing data and materials by including the following statement: "This does not alter our adherence to  PLOS ONE policies on sharing data and materials.” (as detailed online in our guide for authors http://journals.plos.org/plosone/s/competing-interests) . If this adherence statement is not accurate and  there are restrictions on sharing of data and/or materials, please state these. Please note that we cannot proceed with consideration of your article until this information has been declared.  Please include both an updated Funding Statement and Competing Interests Statement in your cover letter. We will change the online submission form on your behalf.  Please know it is PLOS ONE policy for corresponding authors to declare, on behalf of all authors, all potential competing interests for the purposes of transparency. PLOS defines a competing interest as anything that interferes with, or could reasonably be perceived as interfering with, the full and objective presentation, peer review, editorial decision-making, or publication of research or non-research articles submitted to one of the journals. Competing interests can be financial or non-financial, professional, or personal. Competing interests can arise in relationship to an organization or another person. Please follow this link to our website for more details on competing interests: http://journals.plos.org/plosone/s/competing-interests | We note the comment raised with regard to the commercial affiliation of two authors (SD and TL). However, all work completed by SD and TL on the article was completed in the authors’ own time; no support has been provided in any way by the commercial entity for the production of the article. We are happy to acknowledge the commercial affiliation and propose the amended funding statement (below). Please advise if you have any objections to this.  Updated funding statement  “Publication fees for the article are paid by Mott MacDonanld Ltd. The authors received no additional funding for this article. Two of the authors (SD and TL) are affiliated to a commercial organisation (Mott MacDonald Ltd). We confirm there is no commercial affiliation between Mott MacDonald and this article. Aside from publication fees, no funding was provided for the production of the article. The primary data collection (2009 - 2012) for the malaria RDT IRT (Ref: 30) and CEA (Ref: 41) in Afghanistan was funded by the Bill and Melinda Gates Foundation through a grant to the London School of Hygiene and Tropical Medicine. Data on aetiological agents of disease (Ref: 14) was collected with funding from the UK Defence Science and Technology Laboratory. These funding organisations did not play a role in the study design, data collection and analysis, decision to publish, or preparation of the manuscript and only provided financial support in the form of authors' salaries and/or research materials”  Given the concern raised around competing interests in light of the commercial affiliation, we also include a competing interests statement for your consideration.  Competing interests Statement  “The commercial affiliation of two authors (SD and TL) does not alter our adherence to PLOS ONE policies on sharing data and materials.” |

## Response to Academic Editor comments:

| **Comment** | **Response** |
| --- | --- |
| The reviewers made essential comments that need to be addressed before we reconsider this manuscript. For your cost-effectiveness Acceptability Curve (CEAC), please show both interventions within the curve. It will be more informative, even when it takes having three graphs with panels A, B, and C for the perspectives you are interested in presenting. Alternatively, present a Cost-effectiveness Acceptability Frontier (CEAF), which would show only the optimal strategy for each perspective within the same graph. | In line with the suggestion, Fig 7 cost-effectiveness acceptability curve (CEAC) is now split into three panels (one for each perspective), with each panel now showing the probability of both intervention and comparator being the optimal (most cost-effective strategy). |
| Also, report your revised manuscript using the CHEERS guideline and submit a CHEERS checklist along with the updated manuscript. | The checklist has been completed and submitted as a supplementary document to the manuscript. |

## Response to Reviewer 1

***Reviewer #1****: In this study, a decision analytic model was used to examine the cost-effectiveness of POCCRP to improve antibiotic prescriptions in malaria-negative patients in Afghanistan. The study concluded that POCCRP tests could improve antibiotic prescribing in Afghanistan. Overall, this economic evaluation has been well conducted and I have a few comments.*

| **Comment** | **Response** |
| --- | --- |
| The cost-effectiveness of POCCRP testing has been studied in many settings previously. However, not much in a LMIC setting as a result, this study adds a different dimension to the literature on the cost-effectiveness of POCCRP testing. | No response required. |
| The perspective of the study should be made explicit in the abstract. Presently, this is not really clear in the abstract. | The study perspectives have been added into the abstract (see lines 30 - 31). |
| With respect to the outcome measure that has been used, my main concern was the fact that there is no threshold to determine whether the intervention is cost-effective or not. It would be interesting to consider other outcome measures as well e.g. the QALY or the DALY. | The measure of outcome considered in the CEA is the number of febrile malaria-negative patients correctly treated. We accept that a limitation of the study is that we do not have a threshold to determine whether the intervention can be considered cost-effective, as we do not know society’s willingness to pay for a correctly treated patient. We acknowledge this as a limitation in the *Discussion* section.  The study was not designed as a cost utility analysis as it is unlikely that utility tariffs mapped to events in the decision tree model would be sufficiently sensitive to capture the value that patients / health services would place on correct diagnoses. The QALY/DALY benefits would likely come further down the road in terms of avoiding antimicrobial resistance. To fully capture this would require a highly complex decision model which was beyond the scope of the current study. We acknowledge however that this is an important avenue for future research.  We note that QALY/DALY measures of outcome would allow for comparison to other healthcare interventions, and it could possibly allow for greater speculation on whether the intervention would be cost effectiveness using WHO guidelines for cost-effectiveness in LMICs (i.e., considering the ICER against the country’s GDP). However, even that does not necessarily directly reflect society’s threshold for WTP for a QALY gained or DALY averted.  We have amended the discussion section of the paper to explicitly reflect on the need for future research over a longer time horizon to capture the QALY / DALY benefits of reduced AMR (see lines 409 – 416). |
| Great to include the scenario analysis which accounts for the cost of antibiotic resistance. However, it is not really clear how this cost was included in the model. | Supporting information file 2 (S2 POCCRP CEA model parameters and sources) provides more information about this. We have updated the manuscript to make this clearer by including information from S2 to address this point. (see lines 274 - 277) |
| More information on the generalisability of the findings to other settings is needed | We have added a paragraph to the discussion that acknowledges the limited extent to which results are generalisable to other countries. We further note that that further research is warranted in other malaria endemic countries (paragraph starting line 403) . It is important to acknowledge that all health systems are different, with different unit costs and health system organisation. Literature on malaria RDTs shows that their use in clinical practice is varied. We have mentioned in the discussion with regard to various variables that could impact upon cost-effectiveness in different settings (e.g. CRP threshold, adherence to test results, prevalence of bacterial infections in non-malaria febrile patients). We would always recommend locally generated primary data to inform decision making on health policy wherever this is possible. |
| There is an error on line 142 page 14 i.e. last sentence under the heading “POCCRP test intervention and diagnostic accuracy” which needs to be deleted. In addition, page numbers need to be sorted out | The error on page 142 has been deleted. I have checked all the page numbers and they are in good order. |
| Figure 2 is repeated line 161 on page 15. | Corrected. |
| Line 192 is not clear | Sentence has been reworded. |

## Response to Reviewer 2

***Reviewer #2****: This is a well written paper evaluating the cost-effectiveness of a point of care C-Reactive Protein (POCCRP) test for bacterial infection in Afghanistan. A decision model was developed, with a result of a 12% reduction in inappropriate antibiotic prescriptions for POCCRP compared to standard practice, hence potentially reducing the risk of antibiotic microbial resistance in the future, with a cost per additional correctly treated case of $14.33 USD from a health care perspective. I have a number of comments:*

| **Comment** | **Response** |
| --- | --- |
| 1) In the abstract the population size would be helpful to put the results in context. | We respectfully disagree with this suggestion, as we feel the very specific number is distracting in the Abstract. We have instead provided further details in the main body of the text to describe the population in more detail. Here we also explain why the number has been used (see following comment for further information on the population size). |
| 2) In regards to (1) I wasn't sure why the population size of 4391 had been chosen. It was the number of people who happened to be in the trial, but I wasn't sure if this was a useful number for a decision maker. Would this represent the size of an average region? Using a population size of 1,000 though, for example, would make it easier for decision makers to multiple up to their specific region. | The 4,391 population was chosen as this was the number of non-malaria febrile patients from the primary data from the Malaria RDT study. Inclusion criteria from the dataset was a patient diagnosed as malaria-negative after being randomised to either the malaria RDT or clinical microscopy arms of the IRT. This is explained in the Population section (starting line 118) of the Materials and Methods section.  We agree with the reviewer that reporting numbers alone may be misleading / confusing for the reader. We have therefore revised the manuscript to express results in both whole numbers and as percentages. We feel that this now makes the results easier to understand. |
| 3) Overall, I wasn't clear how the data from the trial were used to inform the model other than to be the population selected, given, from what I understood from reading the paper, the trial did not involve POCCRP? Why and how the trial was used could be made clearer. | The reviewer is correct. We did not obtain POCCRP data from the referenced trial. Instead, we use the trial as a single source of information to select the population and provide information on several model parameters. We selected this trial to provide model parameters where possible because it represents a sample of patients with fever that has been diagnosed as malaria-negative and is the best available data in the Afghan setting. Brief details of the cost parameters obtained from the RDT study are provided in the methods section (second last paragraph). Full details of all specific data obtained from the study are explained in more detail in the supporting information file 2 (S2 POCCRP CEA model parameters and sources). Cost parameters taken from the malaria RDT trial were:   - Training to administer and interpret test - Outpatient services delivery cost - Overhead cost of running the pharmacy/dispensary at the healthcare facilities - Patient out of pocket expenditure when patient receives appropriate treatment. (Includes cost of transport, drugs, consultation fees, laboratory fees, or special food) - Opportunity cost of patient’s time when patient receives appropriate treatment - Opportunity cost of carer’s time when patient receives appropriate treatment - Patient out of pocket expenditure when patient receives inappropriate treatment. (Inclusions same as cOPEapp) - Opportunity cost of patient’s time when patient receives inappropriate treatment - Opportunity cost of carer’s time when patient receives inappropriate treatment   In addition to cost parameters listed above, we take the parameter “probability of being prescribed antibiotic under current clinical practice” from the primary data in the cited article.  We have revised the manuscript to provide some further clarification on why this study was used, and signpost to the supplementary appendix where more detailed information can be found. We hope our revisions help to clarify how the trial data were used. |
| 4) On page 14 of the methods, I found the reference standard paragraph a little confusing. I understand diagnostic tests to be tests that are used when making diagnostic decisions regarding a disease. My understanding was the POCCRP are not diagnostic tests but clinical decision making tools to help clinicians decide on the potential best course of treatment (as opposed to screening tools that help identify an increased risk of a disease and hence the need to then be escalated to a diagnostic test). | We accept this comment and have addressed this in the paper to make it clear that the tests are not diagnostic tests as they do not detect bacterial infection, but instead are clinical decision-making tools. We have tried to do this specifically in the Reference Standards section (starting line 145) but also throughout the document. |
| 5) In relation to (4) above, using lab tests as a reference standard for 10mg/L makes sense to me. Using the NycoCard Reader II as a reference does not. To me the NycoCard Reader II sounds like a POCCRP assay device that is used for quality benchmarking. Is this correct? Quality benchmarking is not the same as the actual performance of the device being used, or the same as a reference standard. I might be misreading this whole section, but overall it would help to make clear what device is being used and what performance it has been CE marked at. | For the purposes of this evaluation, we use the NycoCard Reader II as the reference standard to provide information as to how sensitive and specific the POCCRP test is to detect CRP at 10mg/L (data cited from Ref 22). Sensitivity and specificity of the NycoCard Reader are not incorporated into model. We have now made this clearer in the reference standards section. We appreciate that this is a potential limitation and have therefore varied the sensitivity and specificity in the model quite widely (univariate and PSA).  The reference standard that the laboratory is used for is probability a bacterial infection patient has a CRP level of equal or greater than 10 mg/L and that a non-bacterial infection patient has a CRP level of less than 10 mg/L. |
| 6) Table 1: The prevalence of bacterial infection among malaria-negative patients appears to be treated as a probability and not a rate as it should be. If the rate has been converted to a probability this should be made explicit. | When we state prevalence, we are referring to the proportion of malaria negative patients that we would expect to have a bacterial infection at the particular point in time at which they take the test. |
| 7) My biggest concern with the model was that I wasn't clear how the negative impact of missing bacterial cases and not prescribing antibiotics had been incorporated into the model. Although details are given on correct prescribing in the results, no details are provided on the number of bacterial cases missed and no discussion of the potential negative health impact of missing those cases. | We agree with the reviewer. This is a limitation of the study insofar as the model does not capture the impact of treatment incorrectly withheld beyond the current time horizon. The paper incorporates societal costs (household costs) associated with both correct/incorrect decisions by clinicians using the test in a 28-day time horizon as patient costs and patient and carer opportunity costs are accounted when correct and incorrect treatment are administered.  A further line of research would be well advised whether clinicians would be confident in using the tests or whether they highlight the misgivings of the reviews (i.e. likely to ignore test result in the fear of missing a bacterial infection and prescribe antibiotics irrespective of result). We allude this in lines 434 – 437. |
| 8) I wasn't clear why the deterministic analysis was reported as the base case as my understanding is that best practice is to report the probabilistic analysis as the base case given that it directly relates to the assessment of uncertainty. | We have amended the text to remove the deterministic analysis as being described as “base case”. Deterministic and probabilistic analysis are presented on an equal footing. |
| 9) Results page 17 - it would help to have the total population reported right as the start of the results section, particularly given the strange number chosen. | We have added in the population number as suggested (see line 284). |
| 10) Discussion page 23 - "a reduction in unnecessary antibiotic prescriptions of 279 was observed". I wasn't sure what this meant sorry? Is 279 good? 279 per what? There is also no unit given for the denominator in the sentence. Incremental cost per what? | This means that 279 unnecessary prescriptions (12% reduction) were avoided as compared to current clinical practice. We have tried to clarify this in the text more clearly by adding in the denominators for the calculation for the percentage calculations in the manuscript. We cannot make determinations on what the ideal percentage reduction in incorrect prescribing would be, but the greater the better. We instead present the numbers and the output to allow decision makers reach an informed decision. We also use the information in the discussion section to make the comparison of cost per unnecessary antibiotic prescription avoided.  The incremental cost is for the intervention as compared to the current clinical practice and refers to incremental cost per additional correctly treated patient (i.e. data taken from Table 2). |
| 11) Overall, I wasn't convinced by the argument used for the willingness to pay threshold from the US, given that the the US and Afghanistan are polar opposites in many factors. | We accept the point. It is not perfect but there is a lack of reference points for this. We have amended the wording to be clear this is the only reference we could find related to this point. We agree it is not perfect, but believe it is better to include a benchmark and to highlight the lack of research in the area. |
| 12) There was only limited discussion provided regarding if the assumptions made in the model are realistic for if the test was implemented into standard practice. My understanding with POCCRP tests is that a lot of work is required to implement these tests, ensure clinicians use them and to ensure that patients find them acceptable. I didn't feel this was adequately addressed in the paper. There was also no discussion of what model would be used to pay for the assay device - fixed upfront or monthly payments? How was this incorporated into the model? What impact would the cost of the device have on uptake given the limited finances in Afghanistan? | We have tried to clearly outline all of the resource use and parameters for the model (delivery costs include cost of kit, training, outpatient delivery cost, dispensary cost) and the sources we have used (with greater detail provided in supplementary document 2). We believe that this will allow local decision makers to determine if the results are applicable to their specific settings,  We have made the point in the discussion section that the analysis does not take into account clinician decision making (i.e. adherence to the tests) but that further investigation is required to look at the impact in the clinical setting. We believe that commenting on how or where the intervention would be financed is beyond the scope of the study, though we accept it would be an important point that would need further discussion once cost-effectiveness of the interventions has been established. |

## Response to Reviewer 3

***Reviewer #3****: Summary: The grammatical mistakes detract from the description and message, but overall the methods part of this manuscript seems ok. Modelers seem to get more leeway when it comes to their distributional choices and how they account for uncertainty than empirical analyses. I am a little uneasy that so many of the parameter distributions come from a single study. I imagine it's likely that there is not much data from Afghanistan, though it might be nice to at least discuss how these values compare with other places and ensure that those values are included in distributions.*

| **Comment** | **Response** |
| --- | --- |
| Points raised in reviewer summary (above):   a) Modelers seem to get more leeway when it comes to their distributional choices and how they account for uncertainty than empirical analyses b) I am a little uneasy that so many of the parameter distributions come from a single study. I imagine it's likely that there is not much data from Afghanistan, though it might be nice to at least discuss how these values compare with other places and ensure that those values are included in distributions. | a) We followed best practice economic evaluation methodology for the selection of distributions to assign to parameters in the probabilistic analyses. b) We agree that the majority of cost data come from one source. We would argue that this is a benefit of the study as the data come from a published peer reviewed data source and provide the best available data in the Afghan setting. As the model is built from an Afghan payer perspective, we believe that sourcing data from Afghanistan wherever possible is appropriate, and indeed desirable to ensure relevance of the modelling for Afghan decision makers. Single source data are advantageous because they reduce the risk of double counting health state resource usage.   We appreciate that all data sources have limitations. We discuss these issues in the Discussion section and we acknowledge where we believe further research is required into model parameters. |
| 1. There are some grammar and editing mistakes sprinkled throughout this manuscript. At times this is not very serious, but other times it makes the article challenging to understand, e.g. line 170. Manuscripts at PLOS ONE can be rejected based on criterion #5 (The article is presented in an intelligible fashion and is written in standard English). The authors need to read this manuscript more closely. | We have addressed the specific concern cited on line 170.  We have also reviewed manuscript in entirety for grammar/editing errors. |
| 2. (Figure 2) I don't understand the tree diagram. Why is the node for bacterial infection or other cause a circle? The definition in the note for Figure 2 says circle nodes are chance nodes, but I would expect whether or not the person has an infection to be nonrandom. | They are referred to as chance nodes, where there are probabilities attached to each event. Standard economic literature refers to these as chance nodes. We agree they are non-random and confirm that we do not treat them as random. |
| 3. (line 252) One thousand simulations is pretty low. It's questionable whether that allows you to find some of the more rare situations and computing power is pretty easy to come by. I encourage using 10,000 simulations. | In light of the reviewer’s comment, we have investigated whether there was any significant deviation from the PSA results presented with greater number of simulations. When ran the analysis for 1,200 simulations and saw no meaningful difference in results. For example, looking at the healthcare perspective, at WTP of $30, there is a 10.6% chance the POCCRP intervention is optimal with 1,000 simulations. With 1,200 simulations this is 9.7%. Thus, we are confident that it is reasonable to maintain the results as presented based on 1,000 simulations. |
| 4. Was anything done in the simulations to ensure that the probabilities would sum to 1? | Beta distributions give a value between 0 and 1. Within Excel, we programmed the probability of the event not occurring to be 1 minus the sampled probability of an event from the beta distribution, so probabilities on each arm of the model always sum to 1. |
| 5. (line 254) I believe you mean "binary" instead of "binomial". | We accept this point and this has now been edited in the text. |
| 6. (line 262) I didn't understand the WTP range that was chosen for this. In the introduction, the WTP threshold is much higher, though that's per QALY. | CEAC present the probability that an intervention is an optimal use of resource (probability of cost-effectiveness) at a range of different maximum threshold values of willingness to pay to achieve the outcome of interest (i.e. correctly treated patient). The maximum WTP value plotted on the x axis (i.e. up to $60) is the point at which 100% of probabilistic iterations generate an incremental cost-effectiveness ratio below the threshold indicated. We are therefore confident that the modelled CEACs reflect the full range results that would be of interest to decision makers. It is important to note that we are not making any statements with regards to what that threshold WTP value should be. |
| 7. (Figure 3) Why are the values centred around $10? I'm guessing these are the outcome values of the simulations and the range of them. | Yes, this is correct. |

We look forward to hearing from you in due course. Should you have any queries with regard to the submission, please do not hesitate to get in contact.

Yours faithfully,

Simon Dickinson

+44 780 123 9795

[simon.dickinson85@gmail.com](mailto:simon.dickinson85@gmail.com)
